# An RFID-Based Method for Multi-Person Respiratory Monitoring

**DOI:** 10.3390/s22166166

**Published:** 2022-08-17

**Authors:** Chaowei Zang, Chi Zhang, Min Zhang, Qiang Niu

**Affiliations:** 1 School of Computer Science and Technology, China University of Mining and Technology, Xuzhou 221116, China; 2 China Pingmei Shenma Group, Pingdingshan 467000, China

**Keywords:** respiratory monitoring, RFID, frequency extraction, waveform reconstruction

## Abstract

Respiratory monitoring is widely used in the field of health care. Traditional respiratory monitoring methods bring much inconvenience to users. In recent years, a great number of respiratory monitoring methods based on wireless technology have emerged, but multi-person respiratory monitoring is still very challenging; therefore, this paper explores multi-person respiratory monitoring. Firstly, the characteristics of human respiratory movement have been analyzed, and a suitable tag deployment method for respiratory monitoring is proposed. Secondly, aiming at the ambiguity and entanglement of radio frequency identification (RFID) phase data, a method of removal of phase ambiguity and phase wrapping is given. Then, in order to monitor multi-person respiration in a noisy environment, the frequency extraction method and waveform reconstruction method of multi-person respiration are proposed. Finally, the feasibility of the method is verified by experiments.

## 1. Introduction

Respiration is a vital physiological activity of the human body and respiration rate is one of the key vital signs to measure the level of human health [1]. The respiration rate can be affected by a variety of factors, such as physical conditions, emotional changes [2] and even environmental stresses [3,4]. Therefore, respiration rate can reflect a great deal of useful information on human health, and respiration monitoring is widely used in healthcare applications. By installing the respiration monitoring equipment in a smart home, the sleep states of the elderly can be measured at any time [5]. Furthermore, respiration monitoring helps to determine whether the patient is suffering from apnea during sleep and even other diseases such as asthma and chronic obstructive pulmonary diseases [6]. It is also essential to monitor patients’ respiration in hospital wards [7]. In conclusion, respiratory monitoring is a hot research topic with great practical and applied values.

Traditional methods of respiration monitoring are based on wearable devices [8], and the person needs to wear special equipment such as a nose probe [9], chest-belt [10] or waist-belt [11]. Some wearable devices even need to transmit data through wired means, thus, the person can only move in a small area, which is extremely inconvenient. With the rapid development of wireless sensing and mobile computing, researchers have found a new possibility for respiration monitoring technology. If wireless technology is used to monitor a human being’s respiratory condition, the data no longer needs to be transmitted through a wired connection and the person will not need to wear the troublesome monitoring equipment, making respiratory monitoring more convenient and faster. At present, wireless technologies that have explored the direction of respiratory monitoring mainly include Wi-Fi [12], Frequency Modulated Continuous Wave (FMCW) [13], Long Range (LoRa) [14] and RFID [15]. Although the wireless-based respiration monitoring method has been able to achieve the respiratory monitoring of a single human being object, it is still challenging to achieve respiratory monitoring of multiple people simultaneously. The related work of respiratory monitoring based on wireless technology is summarized as follows:

- The Wi-Fi-based respiratory monitoring method uses off-the-shelf Wi-Fi devices, which are low-cost and easy to obtain. Because the Received Signal Strength (RSS) and phase in the Channel State Information (CSI) are sensitive to the tiny displacement of the chest and abdomen when the human body is respiring, the CSI can be used for respiratory monitoring. UbiBreathe [12] achieves respiratory monitoring and apnea detection for a single person using the RSS information in the CSI. In addition, BreathTrack [16] proposed a sparse recovery method to extract the accurate phase information from CSI and used it for respiratory monitoring. Although Wi-Fi-based respiratory monitoring has good respiratory monitoring performance, Wi-Fi generally suffers from a “dead zone” problem [15]. In addition, Wi-Fi is difficult to achieve multi-person respiratory monitoring, because the signals reflected by the human being are mixed together at the receiver and it is difficult to separate them.
- The FMCW-based respiratory monitoring method uses FMCW radar to send out a continuous signal with a linear change in frequency and receive the signal reflected by the human being object. Vital-Radio [17] uses the RF characteristics of FMCW and Fast Fourier transform for respiratory monitoring, but it can only perform single-person respiratory monitoring. DeepBreath [18] establishes a multi-reflected signal interference model and uses independent component analysis (ICA) to achieve multi-person respiratory monitoring. Although the FMCW-based respiratory monitoring can achieve multi-person respiratory monitoring, it has the following limitations: (1) **Cost**. In order to achieve high accuracy, it requires dedicated and expensive hardware to acquire the essential wide frequency band (several GHz). (2) **Computational Complexity**. Blind source separation and independent component analysis [19] need to be solved, and this is an optimization problem. (3) **Deployment**. Antenna arrays need to be deployed at different angles to obtain better coverage and high accuracy.
- The LoRa-based respiratory monitoring method needs to use Universal Software Radio Peripheral (USRP) as a gateway to receive the signal sent by LoRa nodes. The literature [14] uses a signal division method to extract the sensing signal for respiratory monitoring. Due to the low power consumption and long-range characteristics of LoRa communication technology, the system has a long respiration sensing range (over 25 m) and low power consumption. However, due to its own narrowband characteristics, LoRa is difficult to achieve multi-person respiratory monitoring.

Radio Frequency Identification (RFID) [20,21] is a widely used data communication technology that allows contactless communication between the reader and the tag to identify the human being. In recent years, there have been a few works using RFID technology for respiratory monitoring [15,22]. A robust RFID-based RM is presented to estimate accurate respiration rate and detect apnea in dynamic environments [23]. The AutoTag system [24] is proposed as an unsupervised recurrent variational autoencoder-based method for respiration rate estimation and abnormal breathing detection with off-the-shelf RFID tags. However, a few works research multi-person respiration monitoring based on RFID, and LungTrack [15] can only monitor two people’s respiration simultaneously with as many as five RFID tags. Multi-person sensing is a tough and important issue in the field of wireless sensing as signals reflected from different persons are combined at the receiver and it is hard to separate different signal paths for each person. In addition, multi-person respiration sensing is more applicable to real-life scenarios, and it provides benefits to a significant fraction of the population, including couples that share the same bed and new mothers who sleep with their infants [18]. In the paper, we use an RFID reader to query multiple uniquely identified tags simultaneously, and starting from this feature, we can conduct research on multi-person respiratory monitoring. Moreover, due to the lightweight feature of the tag, attaching the tag to the human being does not cause many inconveniences to the person. Instead, this feature can be used to better realize multi-person respiratory monitoring.

The main challenges faced in this paper include the following:

- **Tag deployment issues.** How to deploy the tags to achieve better respiratory monitoring in various scenarios;
- **Data processing and analysis issues.** In the actual environment, there are often random noise and multipath interference, so the data returned by the reader is not accurate. How to process and analyze the data;
- **Tag selection issues.** If multiple tags are deployed on the human being, the reader will return a set of data for each tag. How to select the tags.

The main contributions of this paper consist of the following two parts:

- **Extraction of respiratory frequency and reconstruction of respiratory waveform in a noisy environment.** This paper exploits the characteristics of the raw RFID data and uses data processing methods such as band-pass filtering, wavelet decomposition and differential processing to achieve the extraction of respiratory frequency and reconstruction of the respiratory waveform in a noisy environment. The following two signal processing methods are first proposed by this paper: (1) We found that differencing the extreme value sequence of the respiratory signal after wavelet decomposition and filtering it according to the human respiratory characteristics can effectively extract respiratory information and reconstruct the respiratory waveform. (2) We propose a filtering method that is applicable to method (1).
- **Respiratory monitoring of multi-person.** Most of the current respiratory monitoring research results can only monitor the respiratory of a single person at the same time, while this system can efficiently monitor the respiratory of multiple people at the same time. In addition, compared with other wearable sensors, our approach has the following advantages: (1) **Convenience**. The RFID tag is very small (i.e., sub-${\mathrm{cm}}^{2}$ range), and deploying tags on a human being’s body will not restrict the person’s other activities at all. (2) **Cost**. The wearable sensors used in respiration monitoring are expensive, but the RFID tag is low-cost (i.e., a few cents).

## 2. RFID Underlying Data

**Figure 1:**
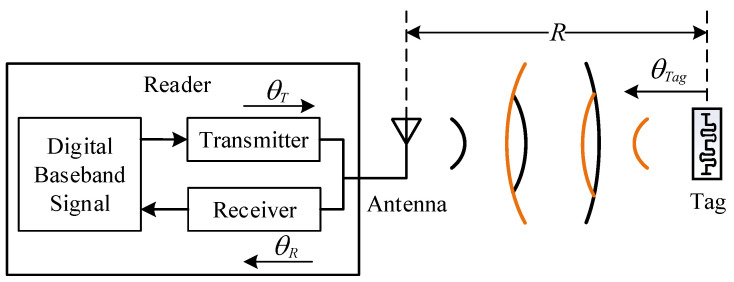
RFID system architeacture.

The RFID system consists of three parts: the reader, the antenna and the tag, the structure of which is shown in Figure 1. The workflow of the RFID system is as follows [25]: First, the digital baseband signal in the reader is modulated and sent out by the transmitter through the antenna. Then, the tag is activated by the signal sent by the reader and uses its own antenna to send out a backscatter signal. Finally, the receiver in the reader receives the backscattered signal from the tag through the antenna and modulates it into a digital baseband signal.

The reader parses the backscattered signal from the tag into readable underlying data, which mainly includes phase, Received Signal Strength Indicator (RSSI) and Doppler Shift. Since the reader used in this paper is Impinj Speedway R420 [26,27], this section is based on the Impinj Speedway R420 reader and introduces the characteristics of each underlying data in detail [25,28].

### 2.1. Phase

Phase is a quantity that describes the variation of the signal waveform. In practice, the transmitter circuit of the reader, the receiver circuit of the reader and the backscattering of the tag introduce additional phase shifts $\theta_{t}$, $\theta_{r}$ and $\theta_{tag}$, as shown in Figure 1. $\theta_{t}$, $\theta_{r}$ and $\theta_{tag}$ can be considered as constant values during one use of the reader. The final phase value θ returned by the reader is related to the signal propagation distance *R* and electromagnetic wave wavelength λ as follows:

(1) $$\theta =2\pi \cdot \frac{2R}{\lambda }+\theta_{t}+\theta_{r}+\theta_{tag}$$

Each time a tag is queried by the reader, the reader returns the phase value of the tag. The Impinj Speedway R420 reader introduces an ambiguity of π radian when processing the phase, that is, the phase value returned by the reader may be the real phase θ or it may be (θ+π)mod2π. The underlying phase field of the Impinj Speedway R420 reader is a 12-bit binary number (0∼4095), each number is mapped to a specific phase value, and the mapping relationship is given by:

(2) $$\varphi =v\times \frac{2\pi }{4096}\left(\mathrm{rad}\right)$$

where *v* is the underlying phase field of the reader and φ is the phase value mapped by *v*. The accuracy of the phase value $\varphi_{res}=2\pi /4096\;\left(\mathrm{rad}\right)\approx 0.0015\;\mathrm{rad}$.

### 2.2. RSSI

RSSI reflects the power measurement value in dBm within the bandwidth of the channel filter. RSSI can be calculated by the power of received signal $P_{R}$ according to Equation (3). In the underlying implementation of the Impinj Speedway R420 reader, the accuracy of RSSI is 0.5 dB.

(3) $$RSSI=10\mathrm{lg}\frac{P_{R}\left(\mathrm{mW}\right)}{1\;\mathrm{mW}}$$

In an ideal environment, the relationship between RSSI and signal propagation distance *d* is shown in Equation (4), where α is the path loss factor and $d_{0}$ is the reference distance.

(4) $$RSSI\left(d\right)=RSSI\left(d_{0}\right)-10\alpha \mathrm{lg}\frac{d}{d_{0}}$$

**Figure 2:**
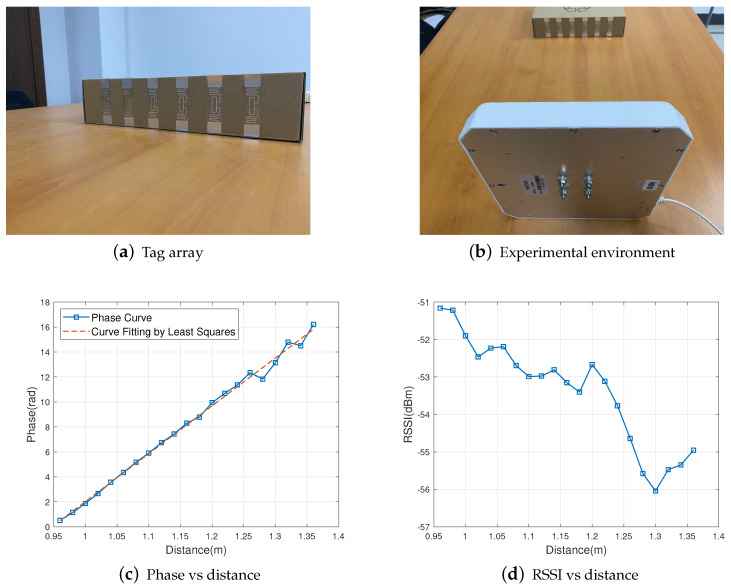
Robustness comparison of phasing and RSSI.

An experiment was conducted in an actual environment to compare phase versus distance and RSSI versus distance. In this experiment, we move six tags from 0.96 m away from the antenna along a straight line to 1.36 m away from the antenna, 5 cm each time, 20 times in total, and measure the phase and RSSI of each tag at each position, the experimental environment is shown in Figure 2a,b. Then, we calculate the average phase and RSSI for each position, and the results are shown in Figure 2c,d. It can be seen that phase and the signal propagation distance still have an obvious linear relationship in the actual environment, satisfying Equation (1). For RSSI, according to Equation (4), RSSI should decrease with the increase of the signal propagation distance, but due to the noise interference from the actual environment, the relationship between RSSI and the signal propagation distance becomes haphazard. Therefore, the phase is more robust to ambient noise than RSSI.

In summary, phase is better than RSSI in terms of numerical accuracy and immunity to interference, so for respiratory monitoring, phase is a better choice than RSSI.

### 2.3. Doppler Shift

**Figure 3:**
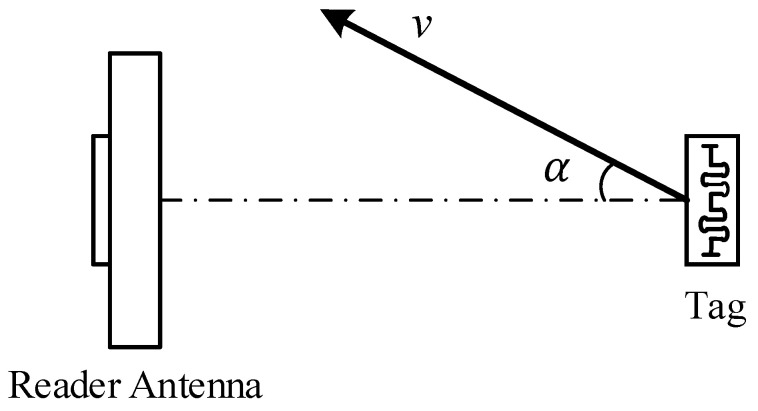
Doppler shift principle.

When the tag is in motion, the frequency of the backscattered signal received by the reader is shifted due to the signal propagation distance difference, as shown in Figure 3. For RFID systems, the Doppler shift $f_{DP}$ is given by:

(5) $$f_{DP}=\frac{2v}{\lambda }cos\alpha$$

where *v* is the magnitude of the velocity of the tag movement, α is the angle between the tag movement direction and the backscattered signal propagation direction, and λ is the electromagnetic wave wavelength.

For the Impinj Speedway R420 reader, the Doppler shift is calculated based on the phase shift Δθ during the packet sending time ΔT, as shown in Equation (6). In the underlying implementation of the Impinj Speedway R420 reader, $\Delta \theta \in \left[4^{\circ },720^{\circ }\right]$, according to Equation (6), it can be deduced that $f_{DP}\in \left[\frac{1}{180\cdot \Delta T},\frac{1}{\Delta T}\right]$. Usually, the data packet sending time ΔT is about 3 ms∼6 ms, so the minimum value of $f_{DP}$ is about 1 Hz∼1.7 Hz. The underlying layer Doppler Shift field is a 16-bit binary number, the first 12 bits represent the integer part, the last 4 bits represent the decimal part, and the unit is Hertz (Hz), so the accuracy of the Doppler Shift $f_{res}=2^{-4}\;\mathrm{Hz}=0.065\;\mathrm{Hz}$.

(6) $$f_{DP}=\frac{\Delta \theta }{4\pi \Delta T}$$

For the Impinj Speedway R420 reader, Doppler Shift cannot be used for respiratory monitoring, here is an example to prove it. The displacement of the chest during breathing is approximately 0.5 cm [29], the human respiratory rate is approximately 12 to 16 breaths per minute [30], and the frequency range of the reader we used is 920.625 MHz to 924.375 MHz [31]. Assuming that the tag is deployed on the chest, the chest is facing the antenna, the displacement of the chest is Δd=0.5cm, the respiratory rate is $f_{r}=0.25\;\mathrm{Hz}$, and the frequency of reader is f=920.625MHz. So the average moving speed of the tag is $\upsilon =2f_{r}\Delta d=1.25\times 10^{-3}\;m/s$, the wavelength is $\lambda =\frac{c}{f}\approx 0.326\;m$ (*c* is the propagation speed of electromagnetic waves, $c\approx 3\times 10^{8}\;m/s$), and the Doppler frequency shift caused by the human breathing motion can be calculated as $f_{DS}\approx 4.02\times 10^{-3}\;\mathrm{Hz}$ according to Equation (5). Because $f_{DS}$ is much lower than the minimum value that the reader can detect, and the Doppler Shift accuracy of the reader is relatively low, the Doppler Shift cannot be used for respiratory monitoring.

### 2.4. Summary

In summary, for the Impinj Speedway R420 reader, firstly, the range and accuracy of the Doppler shift are not suitable for monitoring respiration, secondly, the phase has higher accuracy and better robustness compared to RSSI, so the phase is chosen for respiration monitoring in this paper. The accuracy and range of each underlying data are shown in Table 1.

## 3. Multi-Person Respiratory Monitoring Method

### 3.1. Tag Deployment

A three-dimensional analysis of chest and abdomen movements during respiring in healthy subjects was performed in the literature [32]. It can be concluded that respiratory movements of the chest and abdomen were different for healthy subjects of different ages and genders, and when the healthy subjects were in different positions. Different people have three types of breathing, thoracic breathing, abdominal breathing, and thoracic and abdominal breathing. In summary, it is difficult to cover all conditions with one tag for respiratory monitoring, therefore, we should deploy two tags on both the chest and abdomen of the human being, recording the respiratory movements of the two body parts, which can accommodate all breathing patterns and makes our method more robust.

Due to the inability to establish a correlation between respiratory signals and each human being, existing respiratory monitoring methods based on wireless technology are difficult to achieve multi-person respiratory monitoring, and respiratory monitoring based on lora technology cannot monitor multiple objects at the same time due to its own narrowband characteristics. As the aim of this paper is to explore multi-person respiration monitoring, there are multiple people in the experimental environment and two tags are deployed on each person. To establish the above correlation, it is necessary to identify which body part of which person’s respiratory status is represented by each tag. To solve this problem, we need to map the tag’s Electronic Product Code (EPC) to the person’s number id and the deployed body part bp, that is $\mathrm{EPC}⟶\langle id,bp\rangle$. To establish this mapping, the ID number and the deployed body part are pre-written into the tag’s EPC.

**Figure 4:**
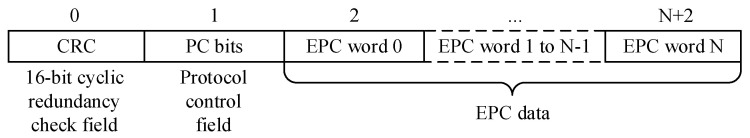
EPC Memory Structure.

**Figure 5:**
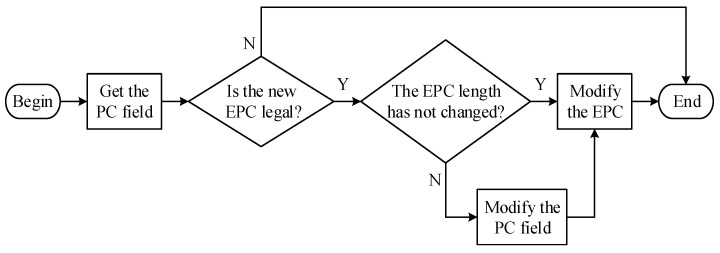
EPC Modification Process.

The Impinj Speedway R420 reader and tag used in this paper adopt the UHF Gen2 standard [33]. Tags under the UHF Gen2 standard are equipped with non-volatile memory. The memory is divided into four independent addressable areas, including Reserved Memory, EPC Memory, Tag Identifier (TID) Memory and User Memory. For  non-volatile memory, 16 bits are 1 word, and each storage area is accessed in units of words [34]. The EPC data that needs to be modified in this paper is stored in the EPC memory. The structure of the EPC Memory is shown in Figure 4, where the Protocol control field defines the data encoding format and the EPC length of the tag. The EPC modification process of the tag is shown in Figure 5, where the legal check includes checking whether the length of the new EPC is greater than the length of the EPC data storage area and checking whether the length of the new EPC is an integer multiple of 16. In addition, if the EPC length is changed, the part (the previous bit) that defines the EPC length in the Protocol control field needs to be reset.

### 3.2. Frequency Extraction and Waveform Reconstruction

**Figure 6:**
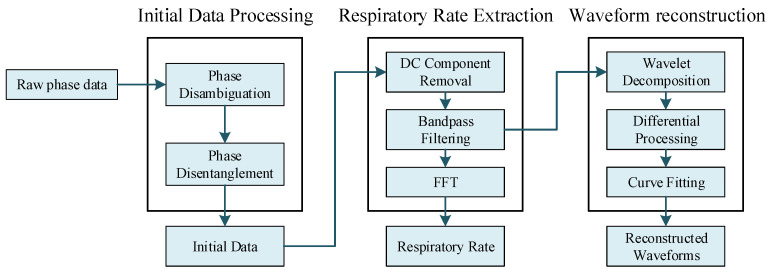
Data Processing Flow.

How to process and analyze the raw phase data returned by the reader and highlight the respiratory status of the human being is an important function of the algorithm. This section will introduce the processing and analysis process of the raw phase data in detail, as shown in Figure 6, which is mainly divided into three parts: data pre-processing, respiratory frequency extraction and waveform reconstruction.

#### 3.2.1. Data Pre-Processing

The pre-processing of the raw phase data consists of two steps, phase ambiguity removal and phase unwrapping, aimed at solving the problem of the phase ambiguity problem introduced by the RFID system hardware and the carrier phase winding problem during signal propagation.

As mentioned in Section 2.1, the phase data returned by the Impinj Speedway R420 reader is introduced with a π radian ambiguity, which will seriously affect the processing and analysis of the phase data in this paper. Therefore, we solve the π radian ambiguity problem first, and the method is shown in Algorithm 1.
**Algorithm 1:** Disambiguation of Phase Data  **input**: Phase data sequence $\Phi =\{\phi_{0},\phi_{1},\cdots ,\phi_{n}\}$, threshold Δ=1
  **output**: Disambiguated phase data sequences $\Phi^{\prime }=\left\{\phi_{0}^{\prime },\phi_{1}^{\prime },\cdots ,\phi_{n}^{\prime }\right\}$

  
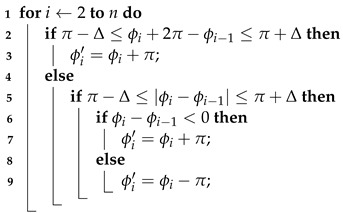



The π radian phase ambiguity is detected based on whether or not there is a π radian jump in the phase value at the current moment, and if so, the π radian phase ambiguity has occurred at the current moment. In this paper, the phase π ambiguity is divided into the following two cases:

(1) The true phase value $\theta_{i}$ at the current moment satisfies $\theta_{i}+\pi >2\pi$, then the phase value returned by the reader at this time is $\varphi_{i}=\left(\theta_{i}+\pi \right)-2\pi =\theta_{i}-\pi$.

(2) The true phase value $\theta_{i}$ at the current moment satisfies $\theta_{i}+\pi \leq 2\pi$, then the phase value returned by the reader at this time is $\varphi_{i}=\theta_{i}+\pi$.

For case (1), it is judged whether the phase data has the π radian ambiguity according to whether the value of $\varphi_{i}+\pi -\varphi_{i-1}$ is within the interval [π−Δ,π+Δ]. If the π radian ambiguity occurs, $\varphi_{i}$ should be $\varphi_{i}+\pi$.

**Figure 7:**
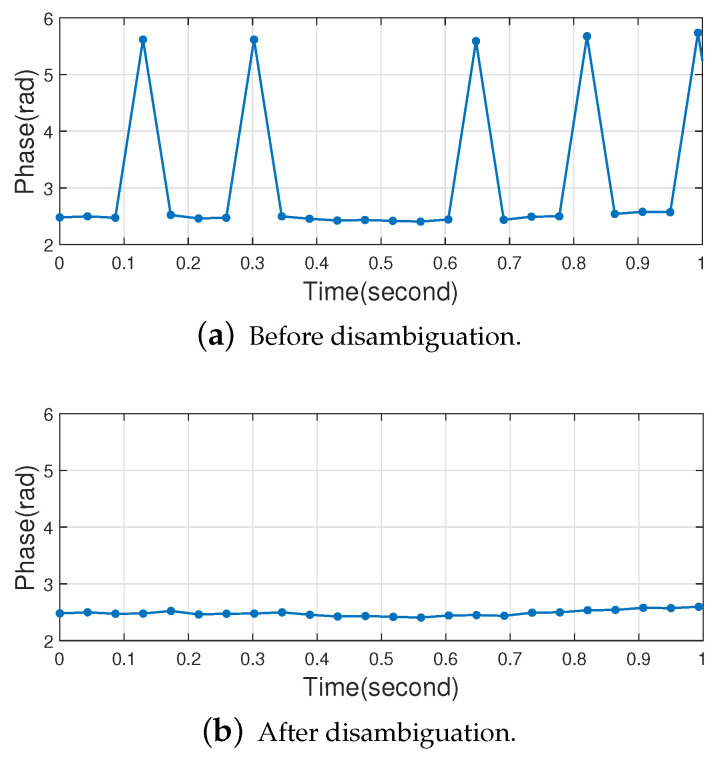
Phase data before and after radian disambiguation.

For case (2), it is judged whether the phase data has the π radian ambiguity according to whether the value of $|\varphi_{i}-\varphi_{i-1}|$ is within the interval [π−Δ,π+Δ]. If the π radian ambiguity occurs, the $\varphi_{i}$ is calculated as follows:

(7) $$\left\{\begin{array}{c} \varphi_{i}=\varphi_{i}+\pi \;\varphi_{i}<\varphi_{i-1}\\ \varphi_{i}=\varphi_{i}-\pi \;\varphi_{i}>\varphi_{i-1} \end{array}\right.$$

where $\varphi_{i-1}$ is the previous moment phase value, in this paper Δ represents a smaller value (threshold) and an appropriate Δ can be selected according to the actual situation. Based on a large amount of empirical results, in this work, Δ is set to 1.8, where the effect of phase ambiguity removal is better at this time. To prove the effectiveness of the algorithm, we used it to filter the actual human respiratory signal in Section 4.2.1 and selected only a small fraction of the result to improve observability. The effect of π radian phase disambiguation is shown in Figure 7.

The second step is to perform phase unwrapping for the phase wrapping problem. The phase wrapping problem refers to the fact that the carrier phase jumps when it reaches the period boundary, thus creating ambiguity. For example, the carrier phase jumps from 2π to 0 or from 0 to 2π. The method for phase unwrapping is shown in Algorithm 2.
**Algorithm 2:** Phase Unwrapping   **input**: Phase data sequence $\Phi =\{\phi_{0},\phi_{1},\cdots ,\phi_{n}\}$, threshold Δ=1

  **output**: Phase sequence after unwrapping $\Phi^{\prime }=\left\{\phi_{0}^{\prime },\phi_{1}^{\prime },\cdots ,\phi_{n}^{\prime }\right\}$

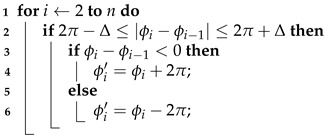


**Figure 8:**
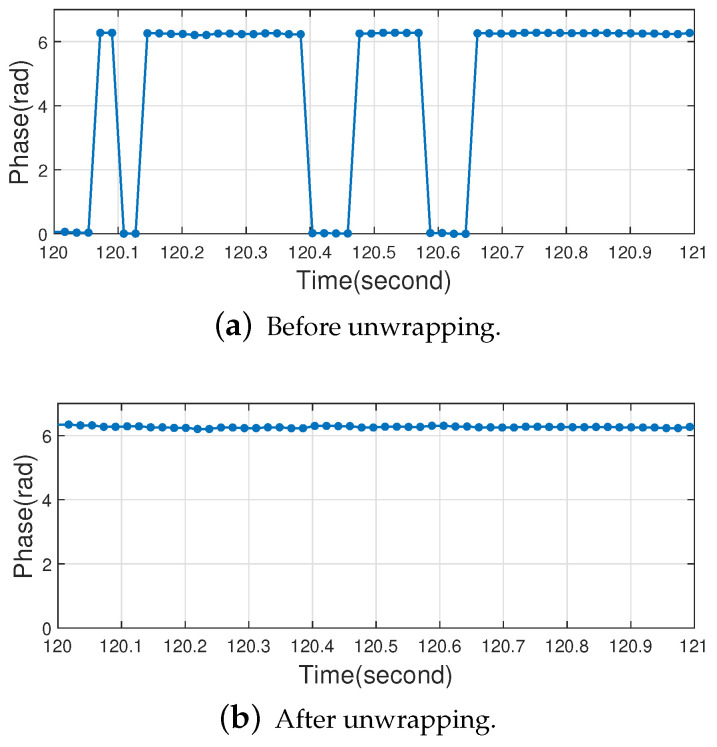
Phase data before and after phase unwrapping.

The idea of the algorithm is as follows. Under the premise that the human body’s breathing motion will not cause a large jump in the carrier phase, when the phase wrapping phenomenon occurs, the phase value $\varphi_{i}$ at the current time and the phase value $\varphi_{i-1}$ at the previous time should satisfy $2\pi -\Delta \leq |\varphi_{i}-\varphi_{i-1}|\leq 2\pi +\Delta$. If the phase wrapping is detected, $\varphi_{i}$ is calculated according to Equation (8) for unwrapping. We also selected a small fraction of the actual human respiratory signal in Section 4.2.1 for testing, and the unwrapping effect is shown in Figure 8.

(8) $$\left\{\begin{array}{c} \varphi_{i}=\varphi_{i}+2\pi \;\varphi_{i}<\varphi_{i-1}\\ \varphi_{i}=\varphi_{i}-2\pi \;\varphi_{i}>\varphi_{i-1} \end{array}\right.$$

At this point, the π radian phase ambiguity problem introduced by the RFID system hardware and the wrapping problem of the carrier phase during signal propagation has all been solved. Now, the pre-processed phase data can be used to extract the respiration frequency and monitor the respiration condition of the human being.

#### 3.2.2. Respiratory Rate Extraction

The standard respiratory frequency analysis is divided into two main types: frequency-domain analysis and time-domain analysis [35]. The frequency-domain performs a Fourier transform of the respiratory signal, and the respiratory frequency estimate is the frequency corresponding to the maximum power in a reasonable respiratory frequency range (4∼60 bpm) [36]. The time-domain analysis identifies reliable breathing as the period of the signal between two pairs of maxima, only one of which is below zero [37]. In this paper, respiratory rate extraction is divided into three steps: DC (zero frequency) component removal, Butterworth bandpass filtering and Fast Fourier Transform (FFT).

**Figure 9:**
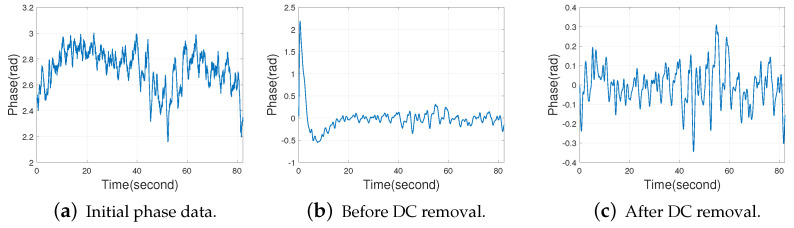
Effect of DC components on 2nd order Butterworth bandpass filtering.

When the reader receives the backscattered signal from the tag, the receiver circuit will introduce a DC component into the respiratory signal [38]. The DC component will affect the subsequent processing of the phase data in this paper. For example, the DC component will cause abnormal values in the initial part of the filtering results of the Butterworth bandpass filter used in this paper as shown in Figure 9, and the waveform in Figure 9a is plotted with the initial phase data returned by the pre-processing of Algorithms 1 and 2. The experimental scenario is shown in Section 4.2.1, the tag is deployed on the chest and the distance between the human being and antenna is 1.8 m. It can be deduced from Fourier transform that the DC component in the breathing signal is the mean value of the signal sequence. Therefore, subtracting the mean value of the signal sequence from each sample value in the signal sequence can remove the DC component in the respiratory signal.

**Figure 10:**
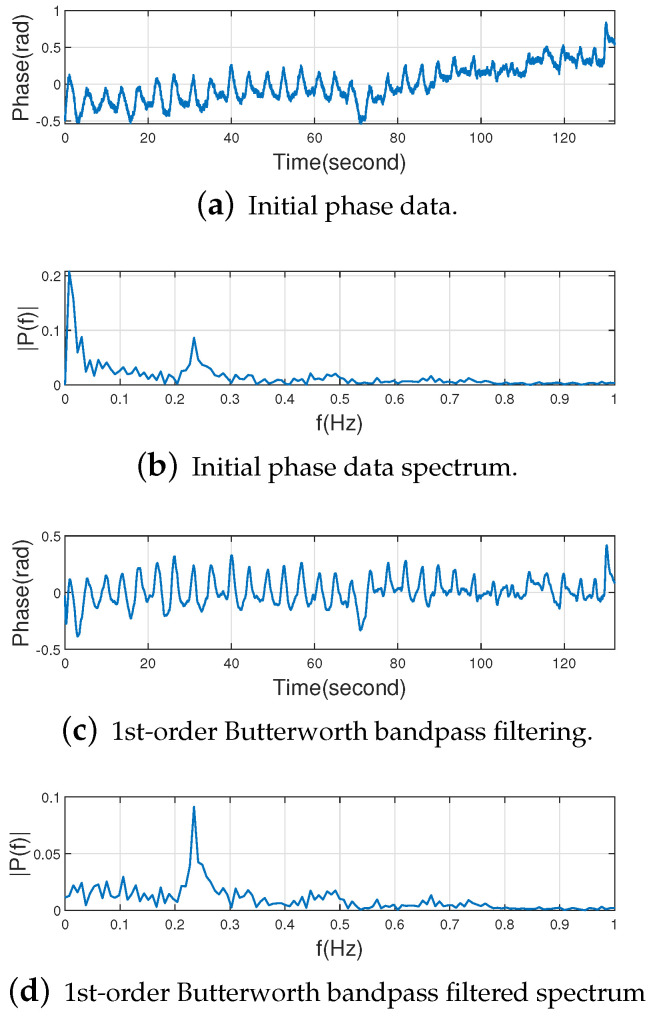

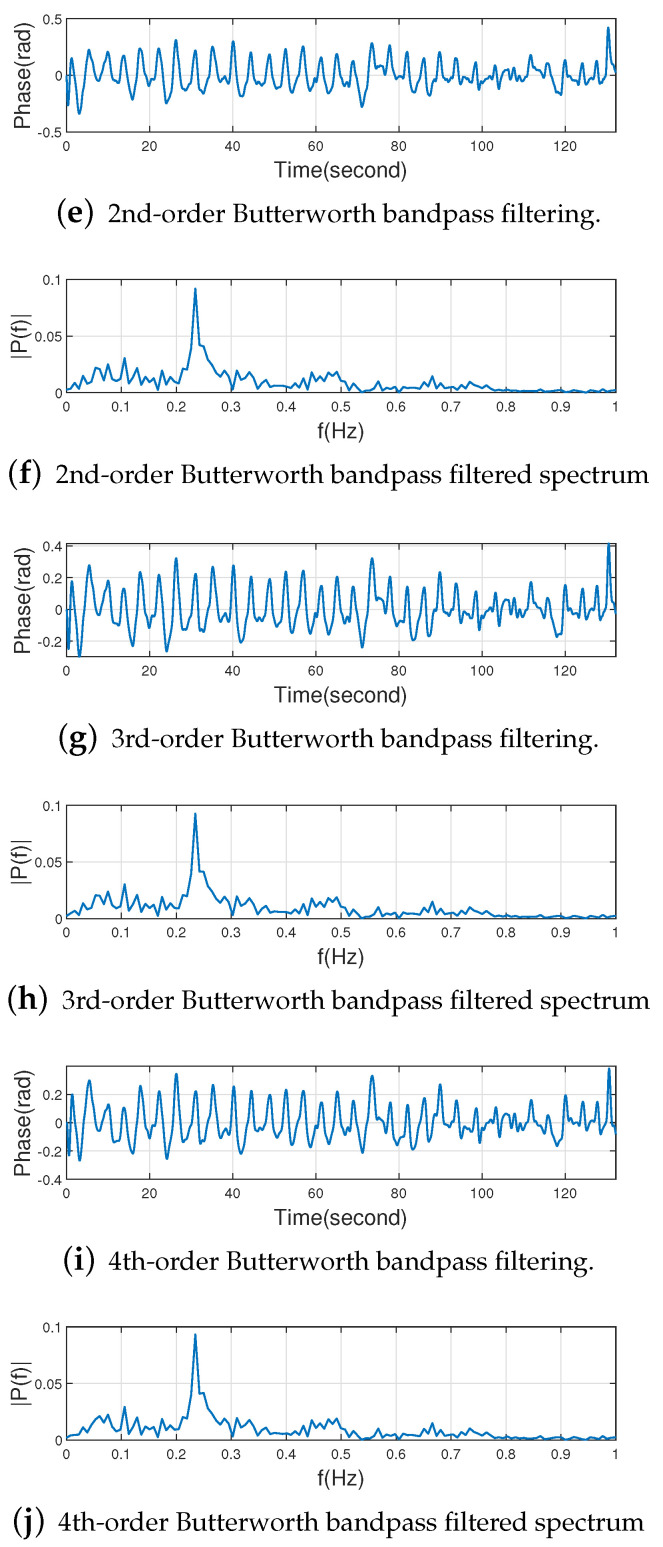
Raw data with 1st to 4th order Butterworth bandpass filtering and corresponding spectrum.

We found in our experiments that although the DC component has been removed, there is still a large amount of low-frequency noise around the DC component. This phenomenon can be seen in Figure 10b, and the initial phase data is also the result of Algorithms 1 and 2. Due to the large low-frequency noise near the DC component, we cannot get the desired result only by removing the DC component. If we want to extract the low-frequency respiratory information, it is necessary to use a band-pass filter to remove both low-frequency noise and high-frequency noise in the respiratory signal. We select the Butterworth bandpass filter as it has a magnitude response that is maximally flat in the passband and monotonic overall. This smoothness comes at the price of decreased rolloff steepness, while Elliptic and Chebyshev filters generally provide steeper rolloff for a given filter order. Since the human respiratory rate is about 0.2 Hz∼0.27 Hz [30], the passband range is set to 0.1 Hz∼1 Hz [15,36] in order to ensure that the spectrum in the respiratory rate range will not be lost. Figure 10 shows the initial phase data, the 1st to 4th order Butterworth bandpass filtering results of the initial phase data and their spectrums.

It can be seen from Figure 10 that the 2nd-order Butterworth bandpass filter has been able to reduce the low-frequency noise in the respiratory signal well and highlight the respiratory rate. Starting from the 5th-order Butterworth bandpass filter, the filtered data will be distorted. In summary, if the order of the Butterworth bandpass filter is too high, the data will be distorted. For the Butterworth filter, the frequency response curve in the passband is maximally flat and ripple-free, while it decreases to zero in the stopband [39]. Moreover, the higher the order of the Butterworth filter, the faster the decay of the frequency response curve in the stopband. As can be seen from the Figure 10d,f,h,j, the frequency response of the respiratory signal within 0 to 0.1 Hz (the cut-off frequency) becomes smaller and smaller as the order of the Butterworth bandpass filter increases. We found in our experiments that the second-order Butterworth bandpass filter can already eliminate the low-frequency noise well enough compared to the first-order Butterworth bandpass filter as can be seen from Figure 10d,f. At this point, increasing the order of the Butterworth bandpass filter again will not result in a significant improvement as shown in Figure 10f,h,j, but will instead increase the computational complexity of the system. Therefore, this paper selects the 2nd-order Butterworth bandpass filter to extract the respiratory rate.

**Figure 11:**
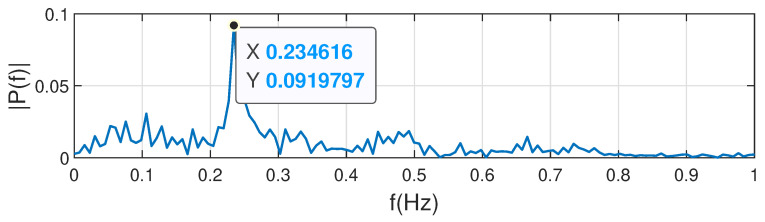
Respiratory rate extraction.

FFT is the main method for extracting respiration rate, and the frequency that corresponds to the maximum power is the respiratory rate we want. To obtain the respiratory rate of the human being, this paper performs FFT on the filtering results of the 2nd-order Butterworth filter. As shown in Figure 11, the frequency 0.234616 Hz with the largest amplitude in the spectrum is the respiratory rate of the human being.

So far, this chapter has completed the extraction of the human being’s respiratory rate through the following three steps: removing the DC component in the respiratory signal, 2nd-order Butterworth bandpass filtering and FFT.

#### 3.2.3. Waveform Reconstruction

In the actual environment, the waveform of the phase data may not be ideal, and it is difficult to display the respiratory state of the human being in real time. This section proposes a method to reconstruct the respiratory waveform, which consists of wavelet decomposition, differential processing and curve fitting.

First, wavelet decomposition [40] is performed on the result of 2nd-order Butterworth bandpass filtering. Wavelet decomposition can effectively remove the high-frequency components in the respiratory signal, extract the low-frequency components of interest in this paper, and analyze the respiratory status of the human being. Wavelet decomposition is to express the signal as a linear combination of wavelet functions in the signal space $L^{2}$, and $L^{2}$ can be written in the following form:

(9) $$L^{2}=\nu_{0}⨁\omega_{0}⨁\omega_{1}⨁,...,⨁\omega_{n}$$

where $\nu_{0}$ is the initial scale function space, $\omega_{0},\omega_{1},\cdots ,\omega_{n}$ are the subspaces from the 0th-order wavelet to the nth-order wavelet tensor, and ⨁ is Exclusive OR function. With each wavelet decomposition, the scale function is reduced by 1 order, and the resolution of the respiratory signal in the frequency domain is reduced to 1/2 of the original. The wavelet decomposition process is given by:

(10) $$s_{\left(t\right)}=\underset{\nu_{0}}{\underset{︸}{\sum_{k}c_{\left(k\right)}\varphi_{\left(t-k\right)}}}+\underset{\nu_{0}⨁\omega_{0}⨁\omega_{1}⨁,...,⨁\omega_{n}}{\underset{︸}{\sum_{j=0}^{4}\sum_{k}d_{j}\left(k\right)2\frac{j}{2}\psi \left(2^{j}t-k\right)}}$$

where $d_{j}\left(k\right)$ is the wavelet function term, φ(t) is the scale function, and ψ(t) is the wavelet function generated by φ(t) and some set of coefficients h(n) defined as follows:

(11) $$\psi \left(t\right)=\sum_{n}h\left(n\right)\sqrt{2}\varphi \left(2t-n\right)$$

**Figure 12:**
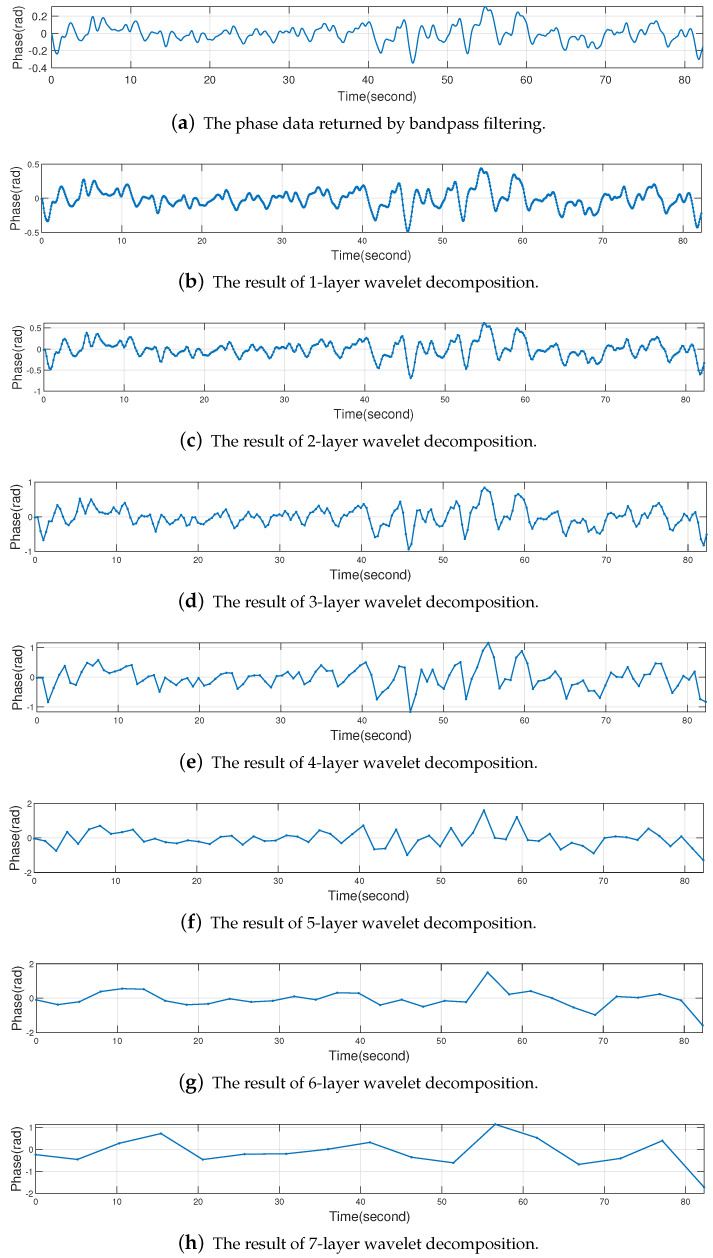
The phase data returned by bandpass filtering vs the results of 1∼7 layer wavelet decomposition.

In RFID technology, the sampling frequency of the reader is affected by a variety of factors, such as the distance between the reader antenna and the tag, the orientation of the tag and the type of tag, etc. The sampling frequency of the reader is not a constant value. In this paper, the number of layers *n* of wavelet decomposition should be selected according to the specific sampling frequency $F_{s}$ of the reader. For the human respiratory signal, this paper has found that the reconstruction waveform is more ideal when the frequency resolution of wavelet decomposition is within 0.6 Hz∼2.0 Hz. Because the human respiratory rate is approximately 12 to 16 bpm [30], in order not to miss the information related to human respiration, we chose a value of 18 bpm (0.3 Hz), which is slightly larger than 16 bpm and easy to calculate, as the lower bound. According to the Shannon Sampling Theorem [41], in order to recover the complete respiratory signal, our sampling frequency $F_{s}\geq 2\times 0.3\;\mathrm{Hz}=0.6\;\mathrm{Hz}$. In addition, in order to prevent the mixing of high-frequency noise, our sampling frequency $F_{s}\leq 2\times 1.0=2.0\;\mathrm{Hz}$ because the human respiratory rate generally does not exceed 60 bpm (1.0 Hz). Therefore, the number of layers *n* of wavelet decomposition should satisfy Equation (12). The calculation of *n* can be deduced as shown in Equation (13), where Z represents the integer field. The reason for taking the maximum value is that we prefer the frequency after wavelet decomposition to be closer to 0.3 Hz (18 bpm) which is a relatively common respiratory rate. For easy understanding, we have given an example in Figure 12, the sampling frequency of the reader $F_{s}=23.1674\;\mathrm{Hz}$ which can be calculated from the timestamps and the number of samples. According to Equation (13), n=5, and the wavelet decomposition result is shown in Figure 12f. When n<5, there is high-frequency noise in the waveform as can be seen in Figure 12a–e. When n>5, the respiratory information will be lost as can be seen in Figure 12g,h.

(12) $$\frac{F_{s}}{2^{n}}\in \left[0.6\;\mathrm{Hz},2.0\;\mathrm{Hz}\right]$$

(13) $$n\in \Omega ,\;\Omega =\left\{e|\left\lceil \log_{2}\frac{F_{s}}{2.0\;\mathrm{Hz}}\right\rceil \leq e\leq \left\lfloor \log_{2}\frac{F_{s}}{0.6\;\mathrm{Hz}}\right\rfloor ,e\in Z\right\}$$

(14) $$n=\underset{n\in \Omega ,\;f(n)\geq 0}{arg min}f\left(n\right),\;f\left(n\right)=\frac{F_{s}}{2^{n}}-2F_{rr}$$

The Daubechies wavelet [42] has better regularity, the smooth error introduced by this wavelet is relatively small. The db1 wavelet (Haar wavelet) does not have symmetry and will produce some phase distortion when performing signal analysis and reconstruction. In addition, the smoothness of the Daubechies wavelet increases as the order increases, but the computational effort increases. Considering the above reasons, we use the Daubechies 2 (db2) wavelet in this paper. The initial phase data returned by bandpass filtering (input for wavelet decomposition) and the result of 5-layer wavelet decomposition are shown in Figure 12. It can be seen that for the 5-layer wavelet decomposition, all the high-frequency details of the respiratory waveform have been removed and only the low-frequency contours are retained. Based on this phenomenon, the idea of reconstructing the respiratory waveform in this paper is to find the maximum values and minimum values of the 5-layer wavelet decomposition result, consider the resulting extreme values sequence as the peaks and troughs of the respiratory waveform, and use this extreme values sequence to reconstruct the respiratory waveform. In Figure 12b, the maximum values and the minimum values of the 5-layer wavelet decomposition result are represented by blue ▴ and red ▾, respectively.

The magnitude of the phase change during a respiratory activity should not be too small. Based on this principle, the current extreme values used to reconstruct the respiratory waveform should satisfy Equation (15), and this paper filters the extreme values sequence accordingly.

(15) $$|e_{i}-e_{i-1}|\geq \alpha \left({\bar{e}}_{\max}-{\bar{e}}_{\min}\right)$$

where $e_{i}$ is the current extreme value, $e_{i-1}$ is the previous extreme value, ${\bar{e}}_{max}$ is the average of the maximum values, ${\bar{e}}_{min}$ is the average of the minimum values, and α is a constant of proportionality (α is 0.5 in this paper).

Then, a differential processing is performed on the extreme value sequence $\Phi =\{e_{0},e_{1},\cdots ,e_{n-1}\}$. The differential processing can reduce the irregular fluctuation of the phase data and highlight the change trend of the phase. The result of differential processing is $D=\{d_{0},d_{1},\cdots ,d_{n-2}\}$, where $d_{i}=e_{i+1}-e_{i}$. In a respiratory activity, for the adjacent peaks and troughs in the phase sequence, the phase value at the peak should be greater than the phase value at the trough. Based on this principle, any two adjacent values of the differential processing results should satisfy Equation (16), and this paper filters the differential processing result accordingly.

(16) $$\frac{d_{i}}{d_{i-1}}=-1$$

**Figure 13:**
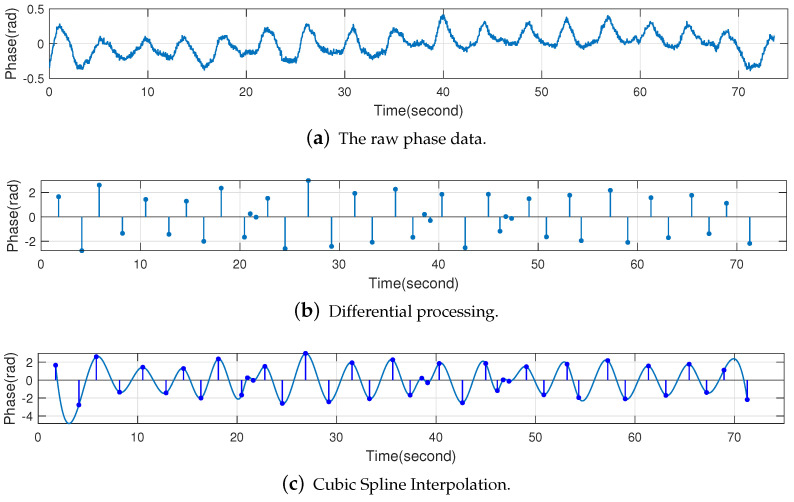
Differential processing and Cubic Spline Interpolation.

Finally, Cubic Spline Interpolations [43] is applied to the result of the differential processing to obtain the final reconstructed respiratory waveform. The final result is shown in Figure 13, which shows that the reconstructed respiratory signal can show the respiratory status of the human being more clearly.

At this point, this chapter has completed the reconstruction of the respiratory waveform of the human being through wavelet decomposition, differential processing and Cubic Spline Interpolation.

### 3.3. Tag Selection

Section 3.1 has introduced the deployment of two tags on each person in this paper, so the reader will return two sets of phase sequences for each person, which leads to the issues of tag selection and processing. This chapter will discuss how the tags are selected and processed.

Assuming that the result of differential processing is $D=\{d_{0},d_{1},\cdots ,d_{n-2}\}$, and *L* represents the total number of elements in *D*. The selection of tags should satisfy the following two principles:

(17) $$2f_{\min}\Delta t<L$$

(18) $$\frac{1}{L}\sum_{i=0}^{L-1}\left|d_{i}\right|>\Delta$$

For the first principle (Equation (17)), the length of the differential processing result should not be too small per unit of time, and it should match the actual respiratory rate of the human body (0.2 Hz∼0.33 Hz). In this paper, $f_{min}$ is set to 0.05 Hz, that is to say, if the monitored respiratory rate is lower than 0.05 Hz, this result will be considered unrealistic and this tag will not be selected in this paper. Δt is the time of respiratory monitoring. $f_{min}\Delta t$ needs to be multiplied by 2, because there will be two extreme values in the phase waveform (a peak and a trough).

For the second principle (Equation (18)), the mean value of the sum of the absolute values of the differential processing result should not be too small, if the mean value is too small, it means that the phase waveform does not have large fluctuations, in other words, the tag does not monitor the respiratory status of the human being. It is possible that this is because the deployment position of the tag is not the main position of the human being’s respiratory movement, then we should check another tag at this time, if the other tag also does not monitor the human being’s respiratory status, this indicates that the person is experiencing apnea and the system should alarm.

## 4. Experiments

### 4.1. Hardware Environment

**Figure 14:**
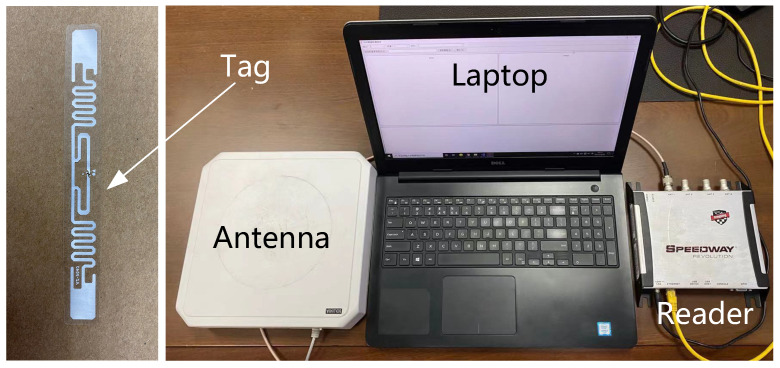
Hardware.

The hardware environment consists of four parts: an Impinj Speedway R420 Reader, some UHF 915M ALIEN AZ-9640inlay long-range passive RFID tags, a VIKITEK VA094 directional antenna for the reader and a laptop with a processor of Intel(R) Core(TM) i7-6500U CPU @ 2.50 GHz 2.60 and 8G of RAM, as shown in Figure 14.

### 4.2. Experimental Results

In this section, the respiratory accuracy Accuracy is calculated based on the Equation (19), where *R* is the actual respiratory rate and $\hat{R}$ is respiratory rate measurement. For ground truth R, one observer counted the number of breaths by visually observing the breathing activities. We also asked the volunteers to self-annotate the number of breaths per minute. If the two records matched one another, we used the obtained number as ground truth [44,45,46]. During the signal acquisition, each sample point $\langle phase,RSSI,timestamp\rangle$ returned from the reader contains timestamp information in milliseconds, and through the timestamps $t_{1}$ and $t_{2}$ of the first and last sampling point, we can calculate the acquisition time as $t_{1}-t_{2}\;\left(\mathrm{ms}\right)$, and the person will note down the number of times he/she respires as *N*. Then, we can calculate the respiratory rate measurement as $\hat{R}=\frac{N}{1000(t_{1}-t_{2})}\;\left(\mathrm{Hz}\right)$

(19) $$Accuracy=1-\frac{|\hat{R}-R|}{R}$$

#### 4.2.1. Effect of Distance on Respiratory Monitoring

**Figure 15:**
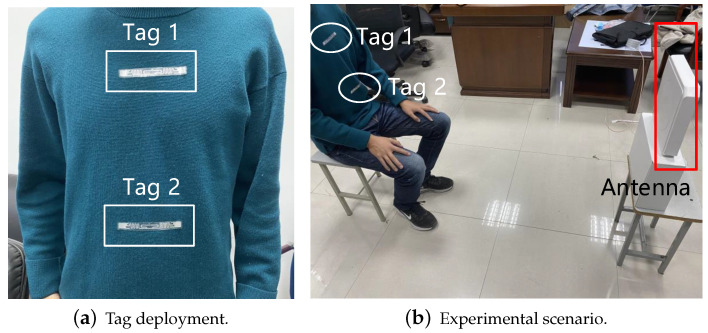
Experimental scenario for the distance comparison experiment.

**Figure 16:**
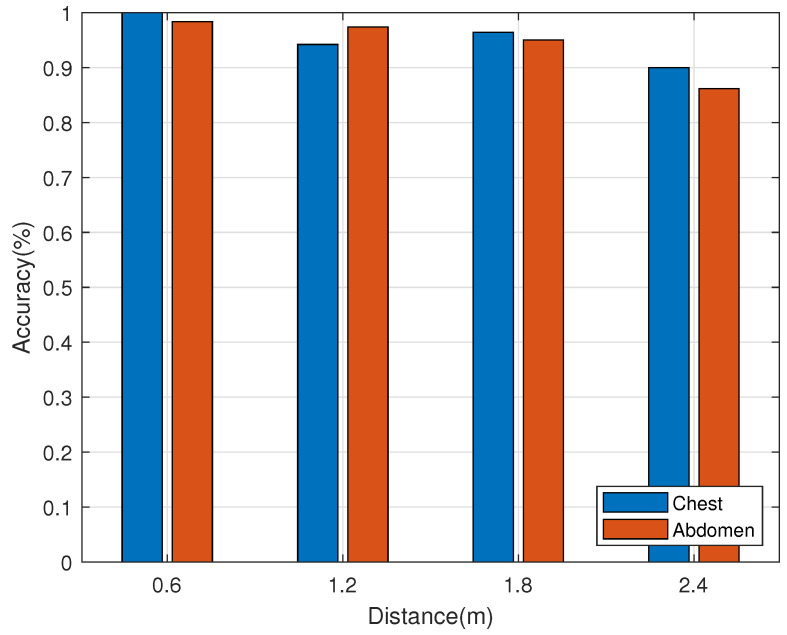
Results of respiratory rate statistics at different distances.

In this section, comparison experiments on respiratory monitoring were carried out at different distances between the reader and the human being, In this experiment, we deployed tags on the chest and abdomen of the human being, respectively, as shown in Figure 15a and the experimental scenario is shown in Figure 15b, the human being is facing the antenna and the distance between the human being and the antenna varies from 0.6 m to 2.4 m, each time by 0.6 m. The transmitting frequency of the antenna is 920.625 Hz. The data during the experiment is shown in Table 2 and the statistical results are shown in Figure 16.

As can be seen from Figure 16, respiratory monitoring is better at distances of 0.6 m, 1.2 m and 1.8 m between the reader and the human being (Accuracy>90%), and the accuracy of respiratory monitoring decreases when the distance is 2.4 m (Accuracy⩽90%). In conclusion, the accuracy of respiratory monitoring decreases with the increase in distance.

#### 4.2.2. Multi-Person Respiratory Monitoring

**Figure 17:**
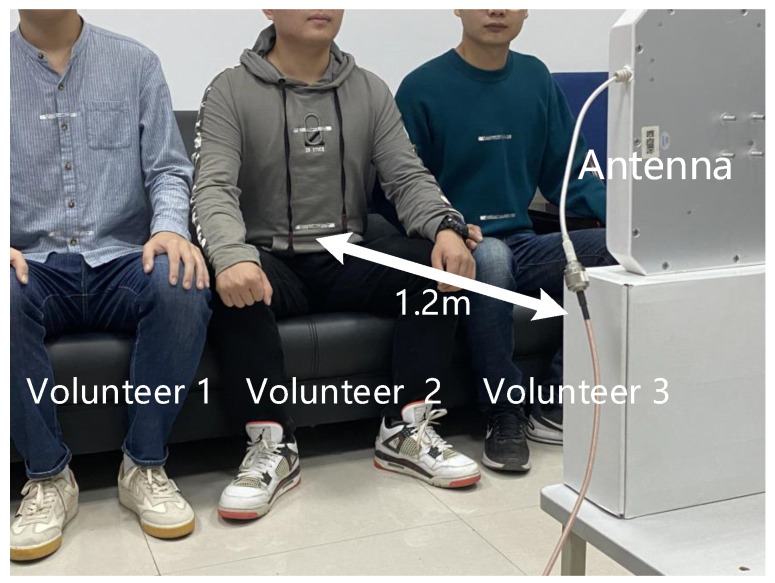
Multi-person respiratory monitoring experiment scenario.

**Figure 18:**
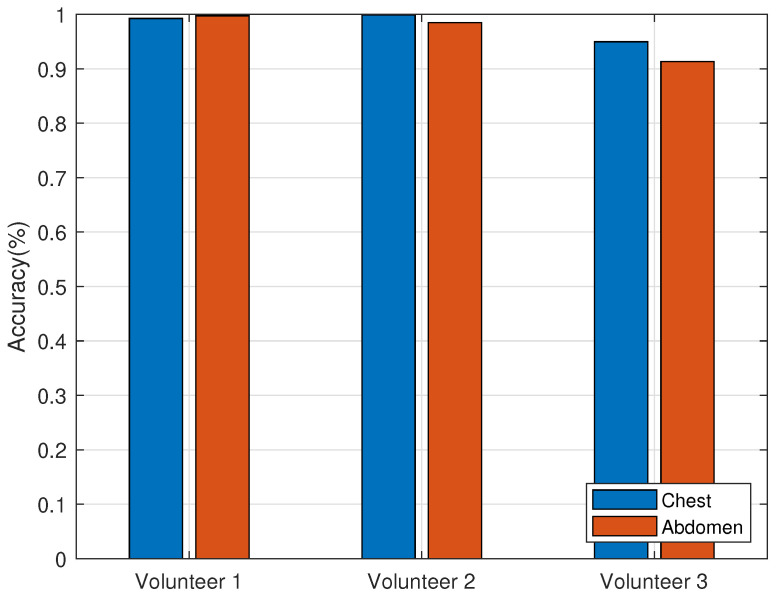
Results of multi-person respiratory monitoring statistics.

In this section, respiratory monitoring was carried out on three volunteers simultaneously, with a distance of 1.2 m between the reader and the human beings. The experimental scenario is shown in Figure 17. Three volunteers sat side by side, facing the antenna, and the tag deployment method is also shown in Figure 15a. The transmitting frequency of the antenna is 920.625 Hz. The data during the experiment are shown in Table 3, and the statistical results are shown in Figure 18.

We also conduct an experiment in a sleeping scenario, where two volunteers are sleeping in one bed with a distance of 0.5 m and the RFID reader is put at the foot of the bed. The accuracy is 92.53% for volunteer A and 94.36% for volunteer B.

As can be seen from the figures, the respiratory monitoring accuracy is greater than 90% and the method in this paper can achieve better respiratory monitoring for multi-person.

#### 4.2.3. Impact of Angular Shift of the Volunteer

**Figure 19:**
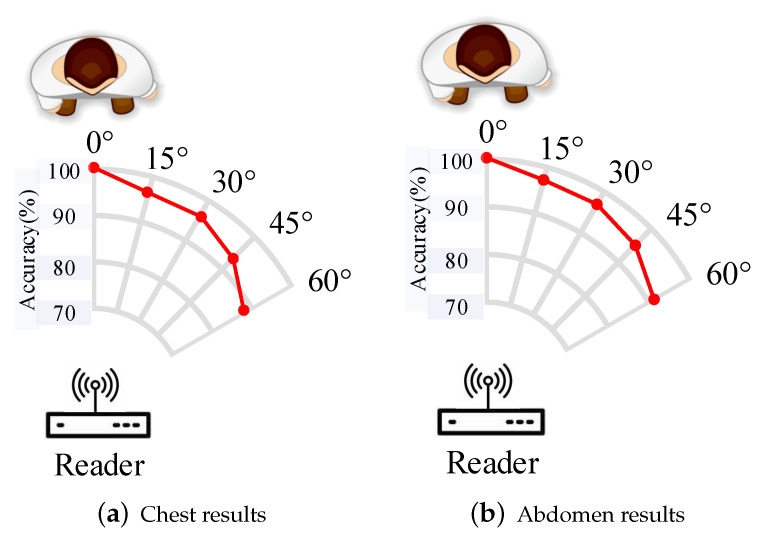
Impact of angular shift of the volunteer.

This section is going to explore the effect of the angular shift of the volunteer on the accuracy of respiration monitoring. We asked Volunteer 2 in Section 4.2.2 to sit in front of the RFID reader with different angular shifts, and the diagram is shown in Figure 19. As mentioned in Section 4.2.2, we obtain an accuracy of 99.25 % from chest variations and an accuracy of 99.37 % from abdomen variations. Figure 19, respectively, shows the accuracies from chest and abdomen variations under different angular shifts. It can be inferred from this figure that with a larger angular shift, the accuracy gets worse. Compared with accuracies when the volunteer and the reader are in a direct line, accuracies decrease 10.02% and 9.17 % when the volunteer is a 60-degree shift from the reader. Experimental results also inspire us to explore the solution of realizing respiration monitoring under different positions in future work.

#### 4.2.4. Apnea

**Figure 20:**
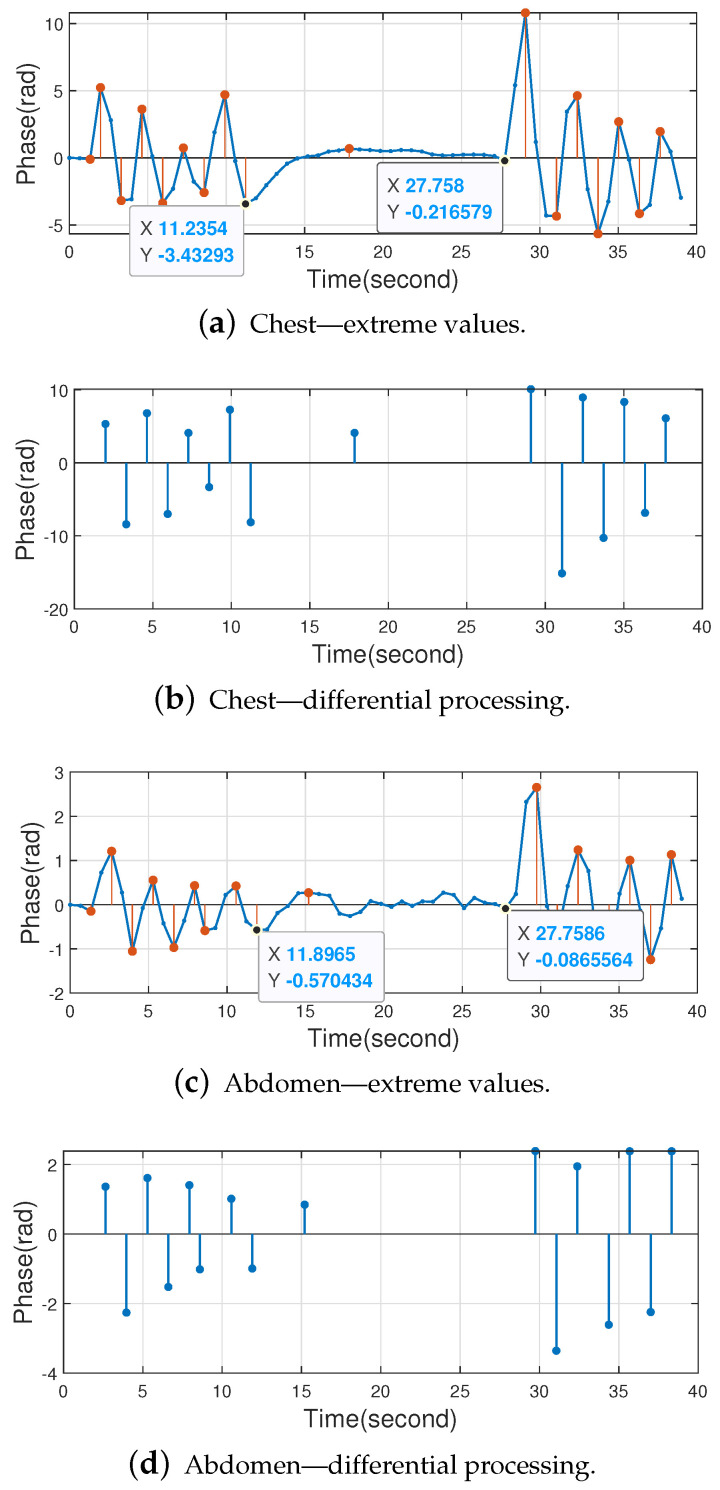
Apnea monitoring using Butterworth bandpass filtering.

This paper explores apnea. The volunteer first breathed for a period of time, then held his breath for a period of time, and finally breathed again. It was found that Butterworth bandpass filtering would have an effect on the results, which are shown in Figure 20.

The extreme value processing results for the chest and abdomen tags are shown in Figure 20a,c. It can be seen that Butterworth bandpass filtering introduces a segment of the abnormal waveform at the beginning of apnea, for example, at the beginning of 11.2354 s to 27.758 s in Figure 20a, and the differential processing results for the chest and abdomen tags are shown in Figure 20b,d. Although Butterworth bandpass filtering causes interference, apnea can still be monitored according to Equation (17), and the calculated results are shown in Table 4.

**Figure 21:**
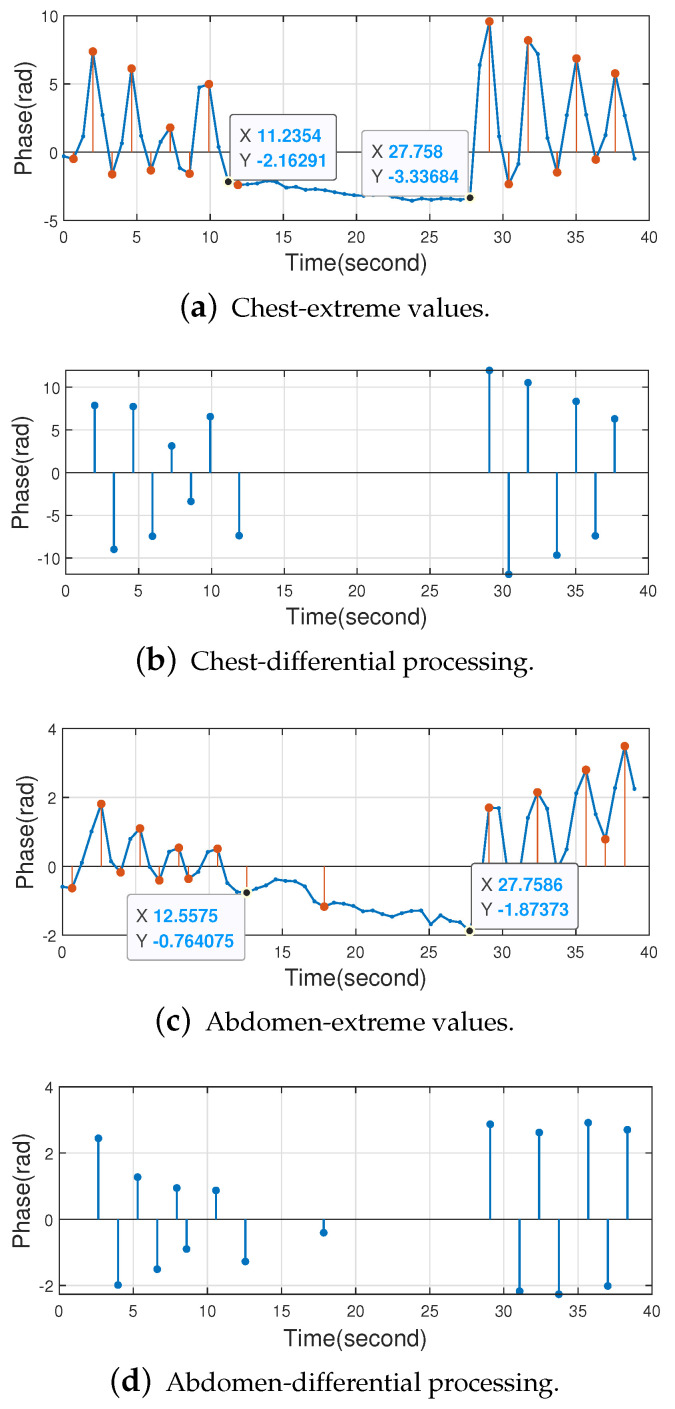
Apnea monitoring without Butterworth bandpass filtering.

Without the Butterworth bandpass filtering, no abnormal waveforms appear at the beginning of apnea, and the experimental results are shown in Figure 21. The calculated results are shown in Table 5. For the chest tag, the number of extremes in the apnea time period is 0 due to the absence of Butterworth bandpass filtering, which is more accurate compared to when Butterworth bandpass filtering is used. For the abdominal tag, although there is one extreme value in the apnea time period due to environmental interference, the apnea condition is monitored.

In conclusion, the method proposed in this paper is able to monitor apnea. For the apnea monitoring function, the performance is better when the Butterworth bandpass filter is not used.

## 5. Discussion

In this section, we briefly discuss the limitations and potential future work.

### 5.1. Non-Los Respiration Monitoring

In our paper, we can achieve multi-person respiration monitoring with RFID technology in an LoS environment. However, the designed approach relies on the direct transmission path from RFID tags to the reader, which hinders its applications in a non-Los environment. In future work, we will further improve our method to make the system suitable for non-LoS respiration monitoring.

### 5.2. Widen Sensing Scopes

The experimental results show that different positions and angular shifts of volunteers may affect the respiration monitoring accuracy. In particular, it can achieve a 60-degree shift with the accuracy of 90.2%, which is a main limitation of the proposed system. In future work, we may explore the placement of RFID devices to enlarge the coverage of good sensing positions. In addition, we can also design a rotatable port for the reader to overcome the limitation of angular shift.

## 6. Conclusions

This paper presents an RFID-based multi-person respiratory monitoring method. Firstly, our design deploys the tags on the chest and abdomen of the human being to monitor respiratory motion guided by the characteristics of human respiratory motion. Secondly, this paper solves the phase ambiguity problem introduced by the RFID reader hardware and the wrapping problem of RFID phase data. Thirdly, we use a 2nd-order Butterworth bandpass filter and FFT to extract the respiratory rate of the human being. Furthermore, this paper uses wavelet decomposition and differential processing to reconstruct the respiratory waveform returned by the 2nd-order Butterworth bandpass filter. Finally, experiments were carried out to verify the validity of our method. We believe our method can provide new ideas in the field of respiratory monitoring.

## Figures and Tables

**Table 1:** Comparison of underlying data.

Underlying Data Name	Data Accuracy	Data Range
Phase	0.0015 rad/$0.088^{\circ }$	$\left[0,2\pi \right]/\left[0^{\circ },360^{\circ }\right]$
RSSI	0.5 dB	$\left[-110\;\mathrm{dBm},0\right]$
Doppler shift	0.0625 Hz	$\left[\frac{1}{180\cdot \Delta T},\frac{1}{\Delta T}\right]$

**Table 2:** Respiratory rate monitoring results at different distances.

Distance	Body Position	Breathing Rate Measurement	Actual Breathing Rate	Accuracy
0.6 m	Chest	0.388803 Hz	0.3889 Hz	99.98%
	Abdomen	0.395226Hz		98.37%
1.2 m	Chest	0.428644 Hz	0.4052 Hz	94.21%
	Abdomen	0.414011 Hz		97.83%
1.8 m	Chest	0.382443 Hz	0.3693 Hz	96.44%
	Abdomen	0.387746Hz		95.01%
2.4 m	Chest	0.403683 Hz	0.3670 Hz	90.00%
	Abdomen	0.417687 Hz		86.19%

**Table 3:** Multi-person respiratory monitoring results.

Volunteer	Body Position	Breathing Rate Measurement	Actual Breathing Rate	Accuracy
2	Chest	0.4073239 Hz	0.4043 Hz	99.25%
	Abdomen	0.4053693Hz		99.73%
3	Chest	0.3367861 Hz	0.3332 Hz	99.87%
	Abdomen	0.3321456Hz		98.47%
1	Chest	0.3350213 Hz	0.3567 Hz	94.97%
	Abdomen	0.3812563 Hz		91.34%

**Table 4:** Apnea monitoring results using Butterworth bandpass filtering.

Body Location	Δt (s)	$2f_{\min}\Delta t$ (Respirating Times)	L (Number of Extreme Values)	Apnea
Chest	16.5226	1.6523	1	✓
Abdomen	15.8621	1.5862	1	✓

**Table 5:** Apnea monitoring results without Butterworth bandpass filtering.

Body Location	Δt (s)	$2f_{\min}\Delta t$ (Respirating Times)	L (Number of Extreme Values)	Apnea
Chest	16.5226	1.6523	0	✓
Abdomen	15.2011	1.5201	1	✓

## Data Availability

Not applicable.
